# Mechanical and biological properties of 3D printed bionic porous Gyroid structure implant

**DOI:** 10.3389/fdmed.2026.1806825

**Published:** 2026-06-02

**Authors:** Xinyu Wang, Yihan Song, Zhixiu Jiang, Zekui Han, Yan Zhang, Zhenyu Song, Yucheng Su

**Affiliations:** 1 Key Laboratory of Microecology-immune Regulatory Network and Related Diseases, School of Basic Medicine, Jiamusi University, Jiamusi, China; 2 Affliated Stomatological Hospital of Jiamusi University, Jiamusi, China; 3 Beijing Implant Training College (BITC), Beijing, China

**Keywords:** bone integration, gradient, Gyroid, implant, TPMS

## Abstract

To investigate the mechanical properties and bone integration capability of bionic gradient porosity Gyroid-structured porous implants. We fabricated porous implants using selective laser melting and large particle sandblasting acid etching techniques, and conducted systematic evaluations through mechanical testing and animal experiments. Mechanical tests demonstrated that the elastic modulus of uniform porosity and gradient porosity Gyroid-structured implants were similar after SLA processing, while the yield strength and compressive strength of gradient porosity Gyroid-structured implants were significantly lower than those of uniform porosity structures. However, all mechanical properties of gradient porosity Gyroid-structured implants matched those of human cancellous bone. Histological examination revealed that at 4 weeks post-implantation, the apical region of gradient Gyroid-structured implants exhibited more new tissue formation compared to uniform porosity Gyroid-structured implants, with no significant difference observed in the neck region. At 8 weeks, bone tissue ingrowth was significantly greater in the neck and apical regions of gradient Gyroid-structured implants compared to uniform porosity Gyroid-structured implants. Additionally, no abnormal inflammatory responses were observed on either type of implant surface. Gradient porosity Gyroid-structured implants demonstrated excellent mechanical properties and enhanced bone integration capabilities, providing a reference for the design of novel implants.

## 1 Introduction

In the 1950s, Professor Branemark proposed the bone integration theory, ushering in a new chapter for dental implantology (1). Since then, dental implant technology has rapidly advanced, and implant prostheses have become one of the primary methods for restoring missing or defective teeth. Currently, all implant materials exhibit excellent biocompatibility (2), including non-toxicity (3), non-allergenicity (4), non-carcinogenicity (5), and non-teratogenicity. Additionally, the mechanical properties, antibacterial performance, and osseointegration capacity of implants can enhance their osseointegration efficiency and prolong the service life of the implants (6). Currently, the most commonly used implant materials are pure titanium (TA1) and titanium alloys (Ti6Al4V, also known as TC4) (7). These materials exhibit excellent wear resistance, corrosion resistance, and good cellular and tissue compatibility, and are widely applied not only in dental implants but also in orthopedic fields. However, these materials have specific limitations: the elastic modulus of titanium and titanium alloys (110 GPa) is significantly higher than that of human bone (0.02–20 GPa), often leading to a stress shielding effect at the implant-bone interface (8). Excessive micro-motion at this interface may result in implant failure (9).

The triply periodic minimal surface (TPMS) is a surface that exhibits periodic variations in three independent directions within three-dimensional space, with zero mean curvature (10), and can extend infinitely in all three periodic directions, providing an accurate descriptive model for various physical structures in both natural and artificial worlds. A representative structure is the Gyroid structure, which can create porous structures with arbitrary numbers of units and volume fractions (11, 12). Compared to traditional point-like structures, TPMS structures inherently possess excellent topological optimization characteristics, offering better self-supporting capability during laser selective melting processes (13). Compared to regular lattice structure scaffolds, the infinitely continuous surfaces with smooth joints reduce stress concentration and enhance mechanical properties (14, 15). TPMS is mathematically defined, and the simple porous scaffold structure obtained by software can effectively overcome the rigidity and design limitations of traditional manufacturing structures (16). This technology can automatically generate digital models of porous bone scaffolds with high-quality surfaces and complex microstructures, enabling precise control over the shape and size of structural units. TPMS materials exhibit an extremely high surface area-to-volume ratio, effectively promoting cell adhesion and proliferation (17, 18). The application of TPMS structures has also been extended to mandibular reconstruction and dental implant design, with promising results in promoting osseointegration (19). With these unique advantages, TPMS modeling technology is regarded as a revolutionary breakthrough in the field of porous structure numerical simulation in the 21st century.

For porous structures, their mechanical properties can be regulated through parameters such as porosity and pore size. Higher porosity and pore size result in lower material strength and elastic modulus, whereas lower porosity and pore size lead to higher material strength and elastic modulus (20). Based on the high density, high strength, and high elastic modulus characteristics of cortical bone, as well as the low density, low strength, and low elastic modulus characteristics of cancellous bone, we adopted a low porosity structure in the cortical bone region and a high porosity structure in the cancellous bone region, ultimately preparing a gradient porous structure (21). This design effectively matches the mechanical properties of cortical bone and cancellous bone (22).

**Figure 1:**
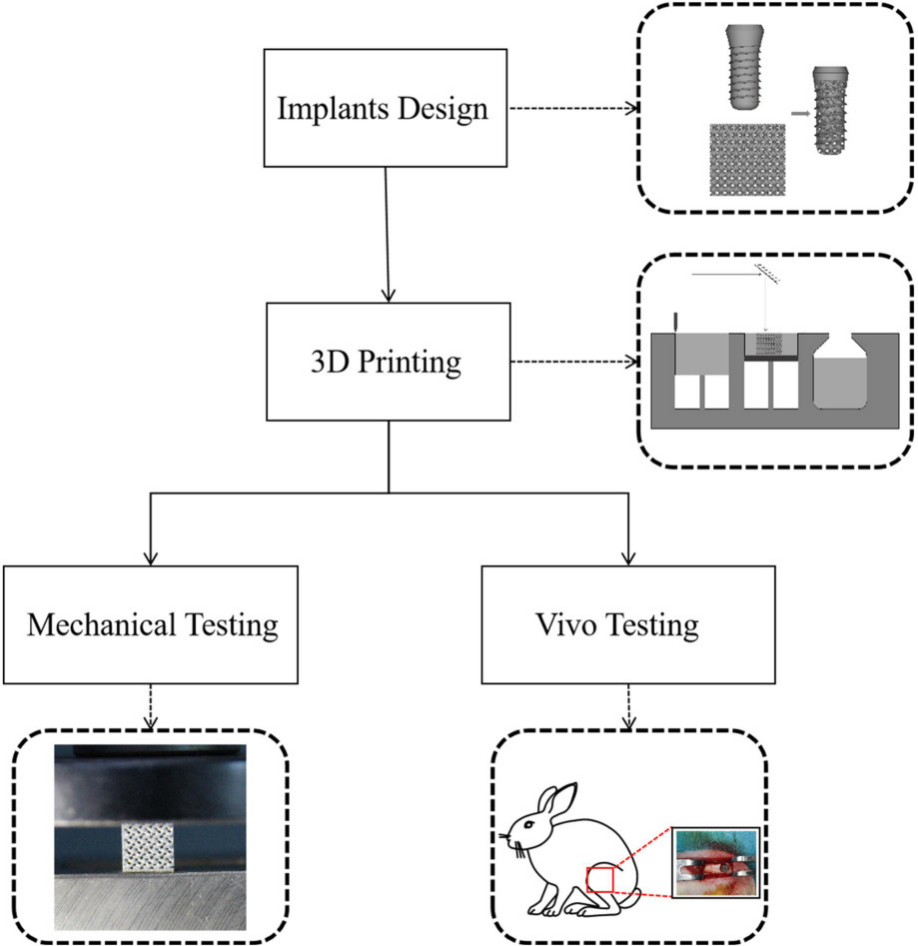
General flowchart of the experiment.

Based on the TPMS-based Gyroid structure, we designed two types of implants: one with uniform porosity (porosity of 70%, h-Gyroid) and another with gradient porosity (porosity gradient of 60%–80%, g-Gyroid). These implants were fabricated using selective laser melting (SLM) technology for Ti6Al4V material (23). After large particle sandblasting and acid etching (SLA) treatment, the mechanical properties of the gradient porosity Gyroid structure implants were evaluated through mechanical compression tests, and their bone integration capability was assessed via animal experiments (Figure 1).

## 2 Materials and methods

### 2.1 Design and fabrication of porous implants

#### 2.1.1 Design of Gyroid-structured implants with uniform and gradient porosity

**Figure 2:**
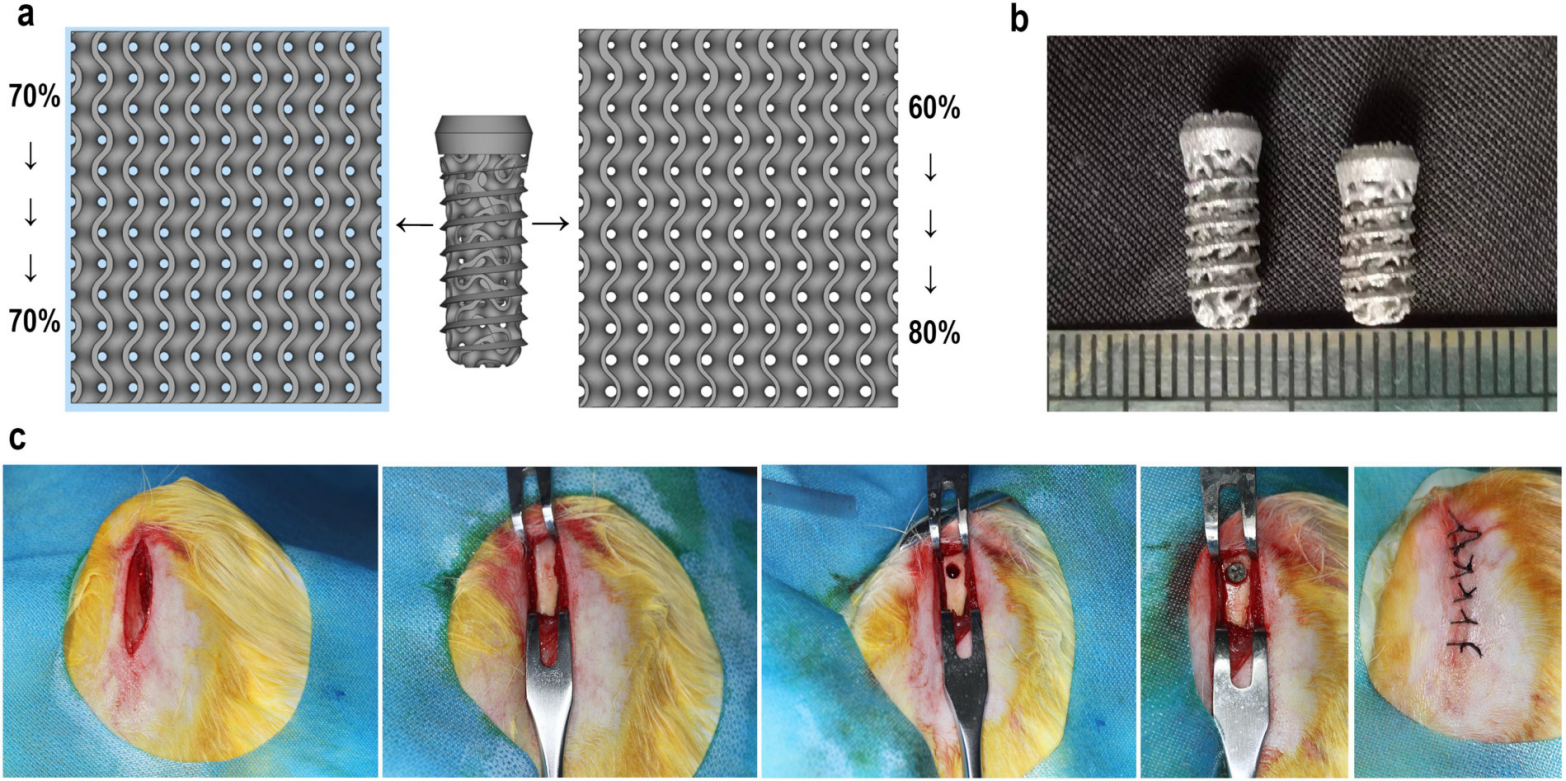
**(a)** design schematic of homogeneous and gradient porosity gyroid-structured porous implants. **(b)** 3D-printed Gyroid-structured porous implants. **(c)** Process flowchart of animal experiments.

In the MSLattice 1.0 software (New York University Abu Dhabi, Abu Dhabi, United Arab Emirates), the Gyroid structure was selected with a cell size of 2 mm, uniform porosity of 70% (h-Gyroid), and gradient porosity of 60%–80% (g-Gyroid), with a mesh density of 30 points (Figure 2a). The data was saved in the standard triangular language (STL) file format. The solid model of the implant was established in SolidWorks 2020 software (Dassault Systèmes SolidWorks Corporation, Waltham, MA, USA). The implant solid model included the cervical segment, porous segment, threads, and central screw. The data was saved in the standard triangular language (STL) file format. The porous implant model was constructed in Magics 19.0 software (Belgium, Materialise). A Boolean intersection operation was performed between the porous segment of the implant solid model and the Gyroid structure. Subsequently, this porous architecture was merged with the cervical segment, threads, and central screw via Boolean union operations, thereby generating the final porous implant model. The resulting model was saved in STL file format.

#### 2.1.2 Preparation and surface treatment of porous Gyroid-structured implants

In the Autofab software(AutoFab MLab64 2.0, Renishaw, Gloucestershire, UK), several support structures with a diameter of 2 mm were generated and exported as a Concept Laser Slicer (CLS) format file. Subsequently, the file was imported into a metal 3D printer for the fabrication of porous implants. The printing parameters were as follows: laser power of 95 W, scanning speed of 900 mm/s, and layer thickness of 0.025 mm. Porous implants and rectangular mechanical specimens were prepared using the Mlab100R laser sintering equipment (Concept Laser, Lichtenfels, Germany), with Ti6Al4V powder (Concept Laser, Germany) of 10–53 μm particle size selected as the printing material. To eliminate stress, enhance the toughness of 3D-printed Ti6Al4V specimens, and improve their mechanical properties, vacuum heat treatment was required with Vacuum Dryer(Hengxin AM Technology Co., Beijing, China). Based on the results of previous exploratory experiments and the surface treatment SLA method (24), the following parameters were selected for the surface treatment of 3D-printed porous Ti6Al4V implants: 60 mesh alumina, 4 cm sandblasting distance, 0.3 MPa sandblasting pressure(Beijing Jiuye Machine Manufacturing Co., Ltd., Beijing, China), controlled temperature of 75°C, reaction time of 30 min, and an acid ratio of 1:1:2 (sulfuric acid, hydrochloric acid, and deionized water). The prepared Gyroid-structured porous implants are shown in Figure 2b.

### 2.2 Mechanical properties of porous implants

Quasi-static compression tests were conducted on porous rectangular specimens using a universal testing machine (Jinan HengRuiJin Testing Machine Co., Ltd., Jinan, China). According to the ISO13314:2011 standard (Standard, 2011) (25), the tests employed porous rectangular specimens with 70% porosity or a 60%–80% gradient structure (dimensions: 12 mm in length, 10 mm in width, with 0.5 mm rectangular plates fixed at both ends to avoid stress concentration during 3D printing and removal of the support structure after printing; 5 specimens of each type). Specimens without SLA treatment and those with SLA treatment were compressed at a rate of 1.2 mm/min. The compression termination conditions were displacement reaching 8 mm or force reaching 99 kN. In the experiment, the elastic modulus (E) was defined as the slope of the stress-strain curve in the elastic deformation region, the offset yield strength (*σ*s) was set as the compression of 0.2% offset stress, and the compression strength (*σ*bc) was the first local maximum in the stress-strain curve.

### 2.3 *In vivo* studies of porous implants

#### 2.3.1 Pre-implantation preparation

In this experiment, a homogeneous porosity (70%) porous implant (h-Gyroid) was used as the control group, while a gradient porosity (60%–80%) porous implant (g-Gyroid) served as the experimental group. After the aforementioned metal 3D printing and surface treatment were completed, high-pressure sterilization was performed. The Poloxamer 407 hydrogel(BASF, Ludwigshafen, Germany) was then prepared by mixing the powder with phosphate buffered saline (PBS) buffer at 4°C and allowing it to stand overnight, resulting in a transparent viscous solution. The prepared hydrogel was placed in a centrifuge tube along with the SLA-treated porous implant and centrifuged at 4°C for 10 min. To prevent inadequate infiltration of blood and exudate into the pores of the porous implant after *in vivo* implantation, we pre-filled the porous implant with Poloxamer 407 hydrogel to avoid the formation of voids that could lead to infection. Finally, the centrifuge tube was preheated in a 37°C incubator for 15 min until the hydrogel gelified, completing the preparation of the homogeneous and gradient porosity Gyroid-structured porous implants for animal experimental applications.

#### 2.3.2 *In vivo* implantation and torque measurement

The animal experiments in this study were approved by the Ethics Committee of the Affiliated Stomatological Hospital of Jiamusi University (Approval No.: KQYXY-2025-ZG-H001). Eight healthy New Zealand White rabbits, aged approximately 8–10 months and weighing 2.0–3.2 kg, were randomly selected. After 2 weeks of acclimatization, the experiment was initiated. General anesthesia was induced via intravenous injection of 20% urethane (50–100 mg/kg) at the ear margin, and the rabbits were immobilized on the operating table after the corneal reflex disappeared. Local anesthesia was administered by injecting an appropriate amount of atropine into the femoral region. A longitudinal incision was made along the femoral condyle, and the bone surface was exposed by layer-by-layer dissection. Low-speed ball drills were used to progressively drill holes at predetermined positions, while saline was used for irrigation and cooling. The prepared homogeneous and gradient porosity Gyroid structure porous implants were implanted into the left and right femoral condyles of the rabbits, ensuring tight apposition with the host bone (Figure 2c). The implantation torque and mobility were monitored during the procedure. After the surgery, soft tissues were sutured layer by layer, and daily povidone-iodine disinfection and intramuscular penicillin injections were administered for 3 days postoperatively to prevent infection.

#### 2.3.3 Histological analysis

Four rabbits were sacrificed at 4 and 8 weeks, respectively, and femoral bones and implants were extracted. The collected specimens were immersed in formalin overnight, and the edges of the bone fragments were trimmed. After fixation for 7 days, the samples were rinsed with water and then dehydrated by immersion in a gradient ethanol solution (50%, 70%, 75%, 80%, 85%, 90%, 95%, and 100% for 3 days each). Subsequently, the samples underwent infiltration for 24 days and were embedded in Technovit 7200VLC resin(EXAKT, Norderstedt, Germany). Sections approximately 30 μm thick were prepared using a diamond-coated saw blade EXAKT 310 CP (EXAKT, Norderstedt, Germany). Finally, methylene blue-acridine pyronin staining was performed. Descriptive analysis and morphometric analysis were conducted on the central portion of the implants. Images were captured using a digital camera (AxioCam MRc, Carl Zeiss, Oberkochen, Germany) of an optical microscope (Axio Imager M2; Carl Zeiss). Morphometric analysis was performed on high-resolution digital images generated by scanning and stitching. Bone-implant contact rate (BIC%), bone volume fraction (BV/TV), trabecular thickness, trabecular spacing, and trabecular number were measured at 1 mm above the neck and 1 mm below the apex of the implant surface.

### 2.4 Statistical analysis

All data in this experiment were expressed as mean ± standard deviation. Statistical analysis was performed using *t*-tests(IBM SPSS Statistics 30, International Business Machines Corporation, Chicago, IL, USA), where *P* < 0.05 indicated a statistically significant difference, and *P* > 0.05 indicated no statistically significant difference.

## 3 Results

### 3.1 Mechanical properties of porous implants

**Figure 3:**
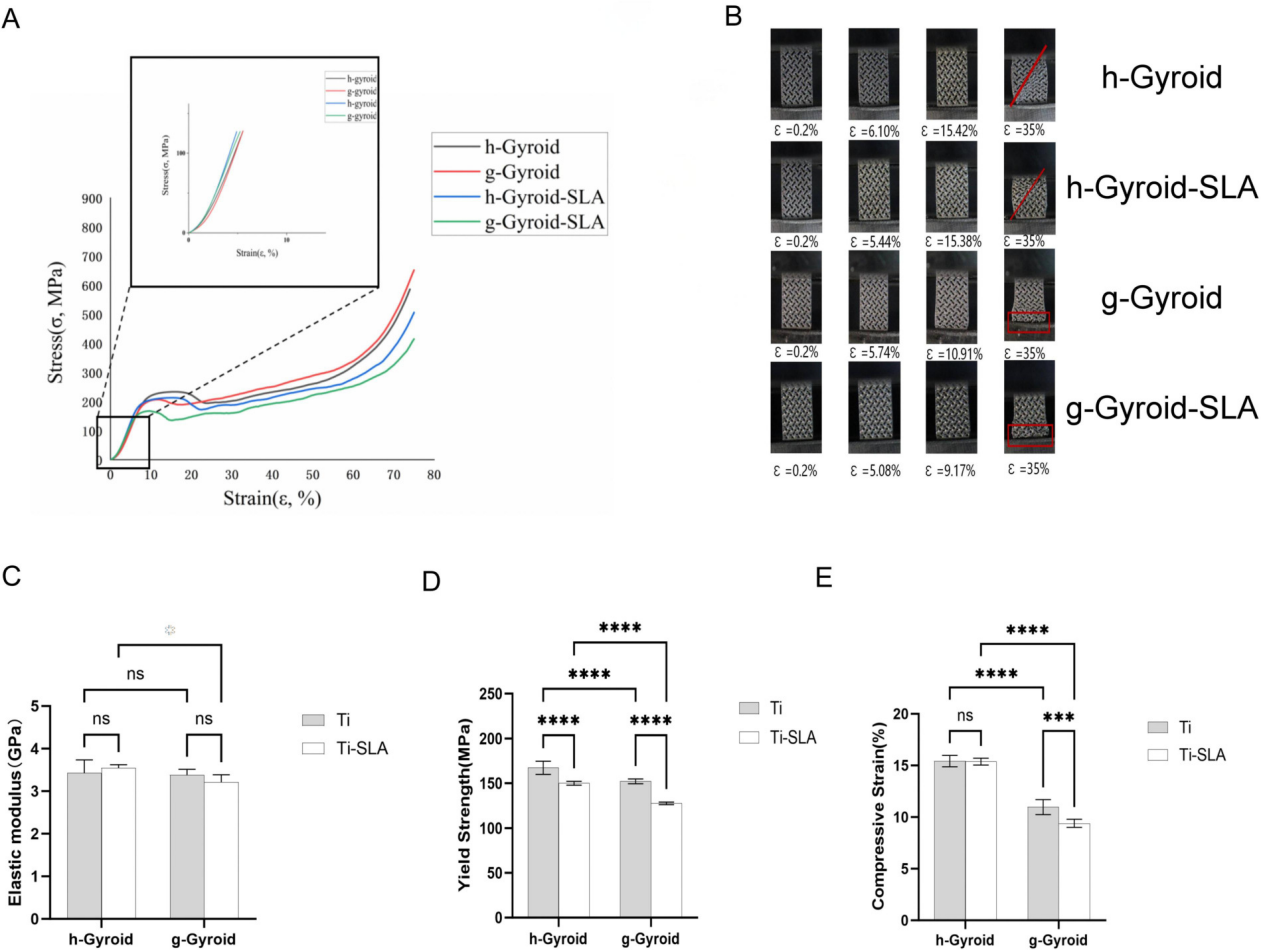
**(A)** compressive stress-strain curves of h-gyroid and g-gyroid structures before and after SLA; **(B)** pictures of the corresponding strain from the compression; **(C)** elastic modulus; **(D)** yield strength; **(E)** compressive strain. Error bars represent means ± SD and specimens number *n* = 4.

Both h-Gyroid and g-Gyroid structures exhibited favorable stress levels before and after SLA processing (Figure 3A). Further observation at the initial compression stage (*ε* = 0.2%), upper yield point (*ε* = 5.08%–6.10%), maximum stress stage (*ε* = 9.17–15.42%), and end of compression stage (*ε* = 35%) revealed evident deformation localization at end of compression stage in both structures before and after SLA processing. Specifically, the h-Gyroid sample fractured along a high-stress zone at a 45° direction (marked in red), while the g-Gyroid sample exhibited progressive localized failure without forming a single dominant fracture plane (Figure 3B). Statistical analysis of the mechanical data for all structures showed that the elastic moduli of h-Gyroid and g-Gyroid samples remained comparable before and after SLA processing, with no statistically significant differences among the four groups (Figure 3C). In contrast, both yield strength (Figure 3D) and compressive strength (Figure 3E) decreased significantly after SLA treatment. The g-Gyroid-SLA samples exhibited lower yield strength and compressive strength than the h-Gyroid-SLA samples, indicating that SLA processing induced a more pronounced softening effect on the g-Gyroid structure, thereby contributing to increased toughness.

### 3.2 *In vivo* animal experiments

#### 3.2.1 Initial stability testing

**Figure 4:**
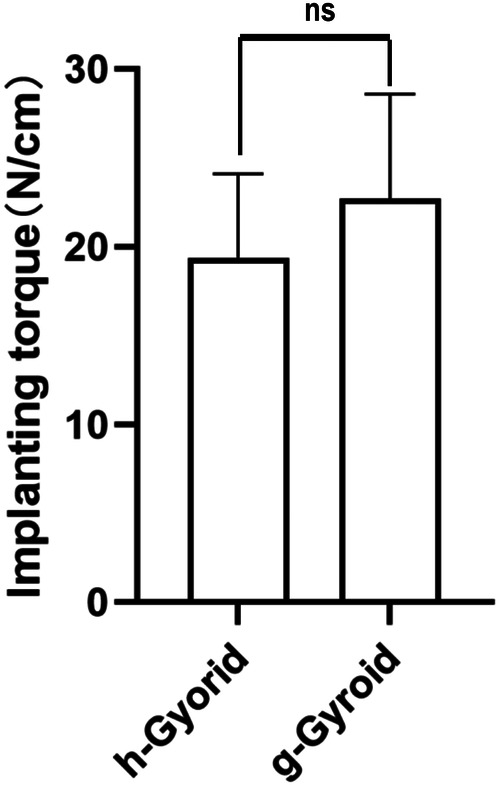
Maximum implant insertion torque (MIT) of h-gyroid implants and g-gyroid implants.

The maximum implant insertion torque (MIT) measured during placement of SLA-processed h-Gyroid and g-Gyroid implants was 19.38 ± 4.73 N/cm and 22.69 ± 5.91 N/cm, respectively (Figure 4). Both values exceeded the commonly accepted clinical threshold for satisfactory primary stability (15 N/cm), indicating that both implant designs achieve adequate initial fixation in rabbit femoral condyles. Although the mean MIT of g-Gyroid implants was approximately 17% higher than that of h-Gyroid implants, statistical analysis revealed no significant difference (*P* > 0.05, Figure 4). This lack of significance may be attributed to the relatively large standard deviations reflecting inter-individual variations in bone quality and surgical placement. Nevertheless, the comparable MIT values suggest that the gradient porosity design does not compromise initial mechanical stability, which is critical for subsequent osseointegration.

#### 3.2.2 Histological analysis

**Figure 5:**
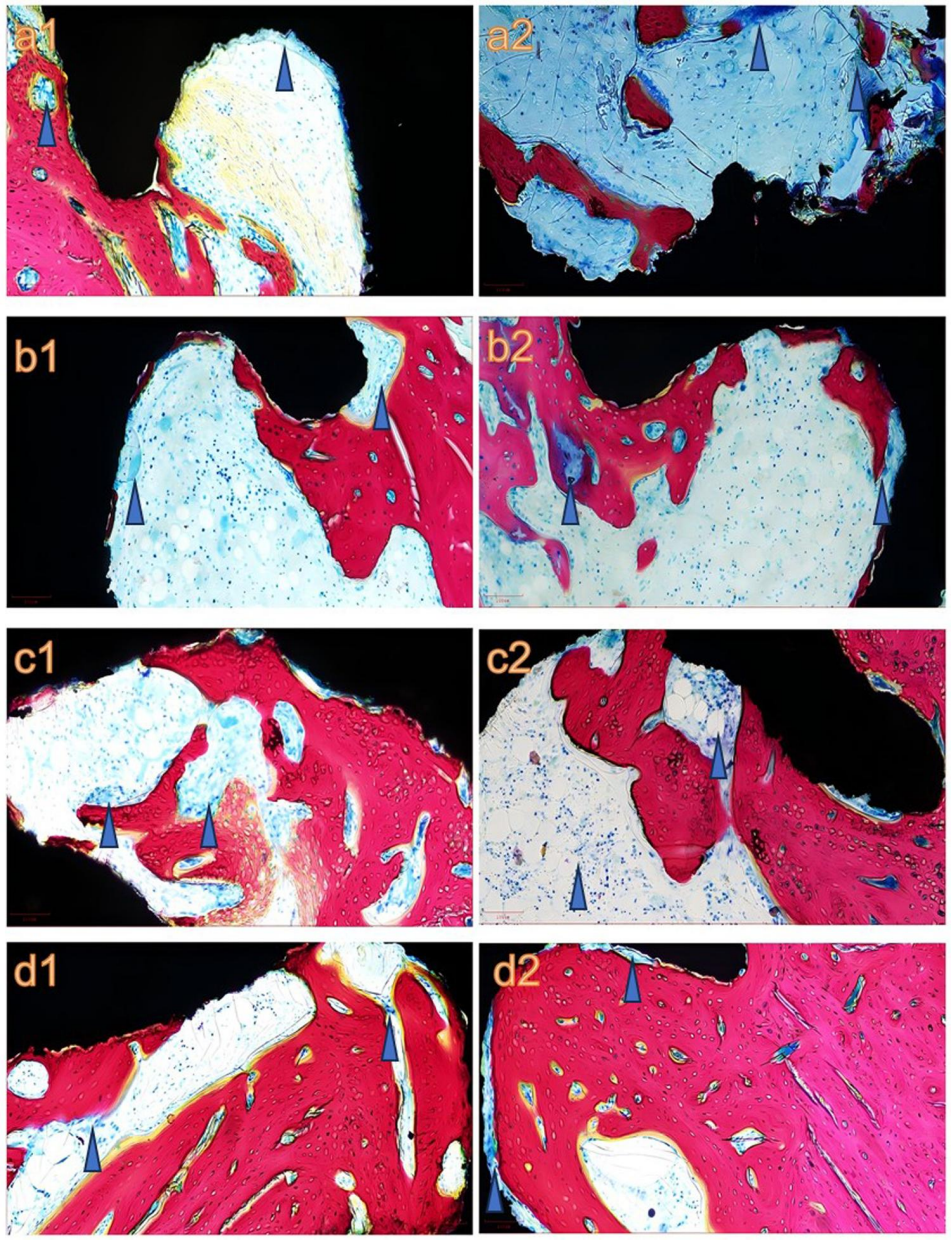
Methylene blue-acridine pyronin staining of bone tissue surrounding the implants. **(a1)** Neck region of h-Gyroid implant at 4 weeks; **(a2)** Root tip region of h-Gyroid implant at 4 weeks; **(b1)** Neck region of g-Gyroid implant at 4 weeks; **(b2)** Root tip region of g-Gyroid implant at 4 weeks; **(c1)** Neck region of h-Gyroid implant at 8 weeks; **(c2)** Root tip region of h-Gyroid implant at 8 weeks; **(d1)** Neck region of g-Gyroid implant at 8 weeks; **(d2)** Root tip region of g-Gyroid implant at 8 weeks. Annotations: Black represents the metal implant, red represents the newly formed bone tissue, and blue spots represent the fibrous tissue. Scale bars = 200 μm.

**Figure 6:**
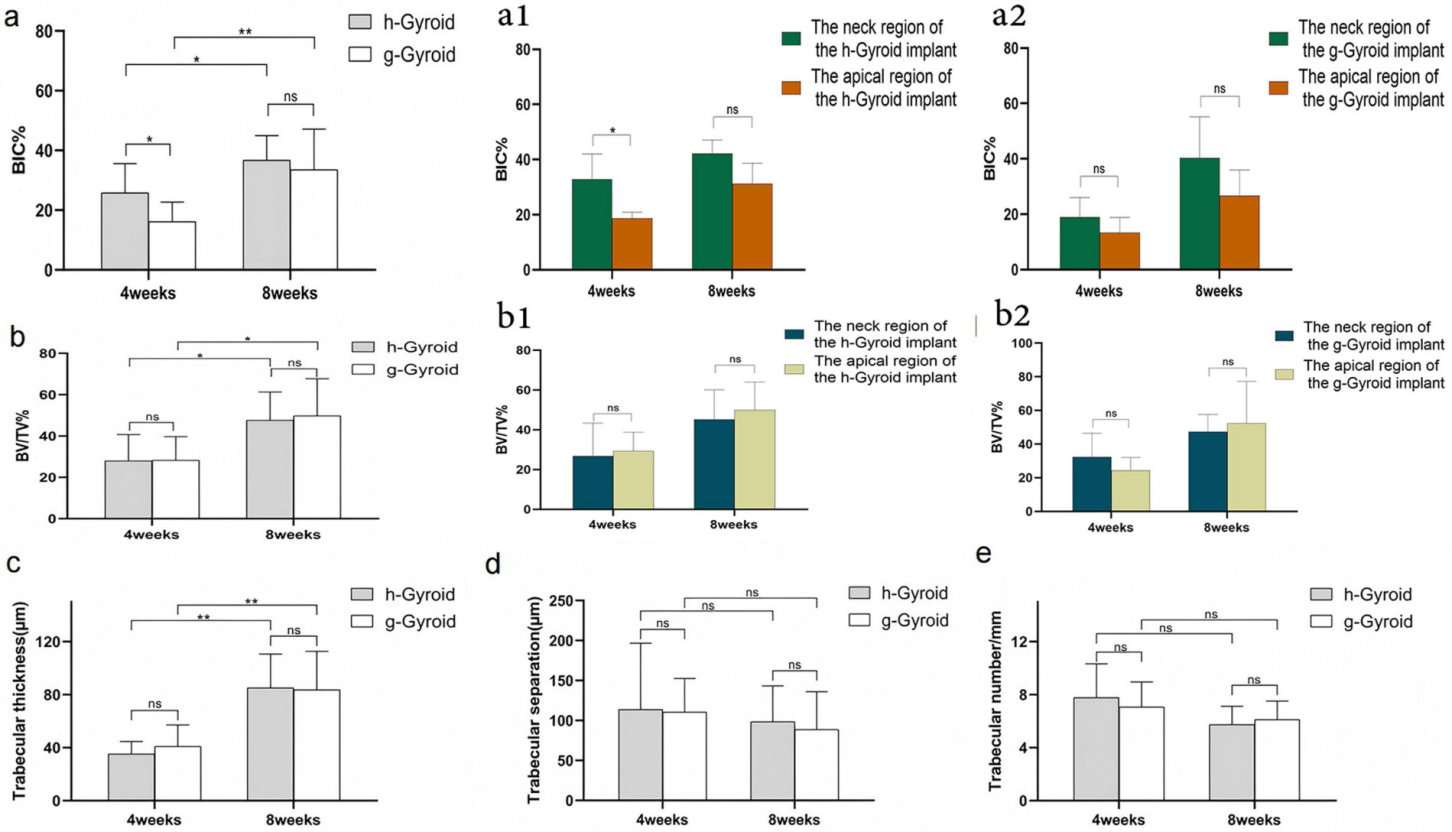
Quantitative analysis of bone-implant integration and trabecular parameters.**(a)** Bone-implant contact (BIC) of h-Gyroid and g-Gyroid implants. **(a1)** BIC in the neck and apical regions of h-Gyroid implants. **(a2)** BIC in the neck and apical regions of g-Gyroid implants. **(b)** Bone volume fraction (BV/TV) of h-Gyroid and g-Gyroid implants. **(b1)** BV/TV in the neck and apical regions of h-Gyroid implants. **(b2)** BV/TV in the neck and apical regions of g-Gyroid implants. **(c)** Trabecular thickness of h-Gyroid and g-Gyroid implants. **(d)** Trabecular separation of h-Gyroid and g-Gyroid implants. **(e)** Trabecular number of h-Gyroid and g-Gyroid implants.

Both h-Gyroid and g-Gyroid implants were successfully implanted in rabbits. At 4 and 8 weeks post-implantation, favorable osseous responses were observed within the implants, with good osseointegration achieved in both the neck and apical regions. Statistical analysis of the newly formed bone tissue showed no significant differences (*P* > 0.05, Figure 6b, Table 1). These results demonstrate that both types of implants exhibit excellent biocompatibility, with no evidence of foreign body inflammation, debris, or fibrous tissue surrounding the implants (Figure 5). Additionally, most of the poloxamer hydrogel in both Gyroid-structured implants underwent degradation.

**Table 1:** BV/TV of h-gyroid implants and g-gyroid implants.

BV/TV	h-Gyorid 4w	g-Gyorid 4w	h-Gyroid 8w	g-Gyorid 8w
Neck(%)	26.80 ± 16.55	32.35 ± 14.07	45.24 ± 14.8	47.27 ± 10.39
Root tip(%)	29.30 ± 9.47	24.28 ± 7.79	50.01 ± 13.95	52.43 ± 24.89
Total(%)	28.05 ± 12.56	28.32 ± 11.38	47.62 ± 13.60	49.85 ± 17.87

At 4 weeks post-implantation, the newly formed fibrous tissue in both types of Gyroid-structured implants was predominantly localized to the central region of the implant, while the bone tissue was mainly distributed at the boundaries of the Gyroid structure. The incompletely degraded hydrogel regions were concentrated in several circular void areas (Figures 5a1,2,b1,2). By 8 weeks post-implantation, histological staining revealed a reduction in fibrous tissue content within the Gyroid-structured implants, with gradual transformation into bone tissue. New bone was also progressively incorporated into the interior of the Gyroid-structured implants, and the hydrogel was almost completely eliminated (Figures 5c1,2,d1,2). Morphometric analysis of the bone regions demonstrated that the bone volume fractions (BV/TV) in the neck and apical regions of h-Gyroid and g-Gyroid implants were 26.80 ± 16.5% and 32.35 ± 14.07%, respectively, compared to 29.30 ± 9.47% and 24.28 ± 7.79%, with no statistically significant differences. As the implantation time extended from 4 to 8 weeks, the bone volume fractions in the neck and apical regions of both types of Gyroid-structured implants increased to 45.24 ± 14.8% and 47.27 ± 10.39%, respectively, and 50.01 ± 13.95% and 52.43 ± 24.89%, respectively, with no statistically significant differences (*P* > 0.05, Figures 6b1,2, Table 1).

Additionally, the bone-implant contact rate (BIC%) at 4 weeks was significantly higher in the h-Gyroid implant group compared to the g-Gyroid implant group (*P* < 0.05, Figure 6a, Table 2), while no significant difference was observed between the two groups at 8 weeks (*P* > 0.05, Figure 6a, Table 2). As the implantation time extended from 4 to 8 weeks, the BIC in both the neck and apical regions of the h-Gyroid and g-Gyroid implants increased significantly and showed statistical differences (*P* < 0.05, Figures 6a1,2, Table 2).

**Table 2:** BIC of h-gyroid implants and g-gyroid implants.

BIC	h-Gyorid 4w	g-Gyorid 4w	h-Gyroid 8w	g-Gyorid 8w
Neck(%)	32.88 ± 9.18	18.90 ± 7.15	42.17 ± 4.94	40.36 ± 14.81
Root tip(%)	18.62 ± 2.29	13.37 ± 5.48	31.30 ± 7.41	26.70 ± 9.27
Total(%)	25.75 ± 9.82	16.14 ± 6.60	36.73 ± 8.23	33.53 ± 13.57

Furthermore, there were no significant differences in trabecular bone thickness, trabecular bone spacing, or number between the h-Gyroid and g-Gyroid implants at all time points (*P* > 0.05, Figure 6c–e). However, the trabecular bone thickness at 8 weeks was thicker in the h-Gyroid group compared to the g-Gyroid group (*P* < 0.05, Figure 6c, Table 3).

**Table 3:** Trabecular feature of h-gyroid implants and g-gyroid implants.

Trabeculae	h-Gyorid 4w	g-Gyorid 4w	h-Gyroid 8w	g-Gyorid 8w
Trabecular thickness(μm)	35.37 ± 9.24	40.89 ± 16.25	85.14 ± 25.52	83.73 ± 28.94
Trabecular separation(μm)	113.90 ± 82.61	110.36 ± 42.20	98.34 ± 44.92	88.63 ± 47.39
Trabecular number(1/mm)	8 ± 3	7 ± 2	6 ± 1	6 ± 1

## 4 Discussion

With the advancement of 3D printing technology, the precision and controllability of products have been significantly improved, leading to the increasing application of complex porous structures in orthopedic implants (17, 20). Moreover, the surface curvature of porous scaffolds plays a critical role in determining tissue regeneration rates (26, 27). Compared to convex surfaces, bone tissue tends to grow on concave surfaces, and the presence of surface tension prevents tissue from entering convex surfaces (28). This implies that lower curvature results in higher bone tissue regeneration levels. Gyroid structures, due to their excellent self-supporting properties, high connectivity, and customizable control, are widely used in implant research. Studies have shown that Gyroid structures fabricated using selective laser melting (SLM) technology exhibit porosities approximately 15% lower than the designed values (29). 3D-printed porous materials often exhibit variations in size and geometry, particularly when manufactured with minimum dimensions near the technical limits (30). This is because the melt pool edge is slightly larger than the laser scanning path, leading to increased wall thickness and residual unmelted particles on the specimen surface (31). In this study, surface treatment using large particle sandblasting and acid etching was employed to achieve porosities close to the designed values (32, 33).

The fundamental properties of implants should be compatible with human biomechanics. For metal implants to serve as substitutes for natural tooth roots, their mechanical properties must first match those of normal bone. We employ computer-aided design (CAD) methods to control the spatial distribution of non-uniform porosity. In the Gyroid-based model, gradient variations in porosity and pore size can be achieved by adjusting constants in the function (34, 35). This unique design approach enables porous Gyroid implants to mimic the hierarchical structure of natural bone, such as the outer dense cortical bone and the inner honeycomb-like cancellous bone (36). Studies have reported that the elastic modulus and compressive strength of Gyroid structures decrease with increasing porosity (37). Increased porosity reduces the wall thickness of the porous Gyroid units, thereby decreasing the solid portion of the specimen and consequently lowering compressive strength. It is well-established that the elastic modulus of human cancellous bone ranges from 4 to 30 GPa (38, 39). To match the elastic modulus of cancellous bone, we aim to select high porosity (approximately 80%) in the cancellous bone region. However, the maximum compressive strength of 80% porosity Gyroid structures is lower than that of human bone tissue (180.5–211.1 MPa) (40), failing to meet implantation requirements. Therefore, we designed and fabricated gradient Gyroid structures (g-Gyroid), which significantly enhance the maximum compressive strength, meeting both implantation strength requirements and compatibility with the elastic modulus of cancellous bone. Meanwhile, the elastic modulus of human cortical bone ranges approximately from 0.2 to 2.0 GPa. The gradient Gyroid structure with 60% porosity applied to the cortical bone can also meet its elastic modulus requirements. Therefore, mechanical experiments have confirmed that the elastic modulus, compressive strength, and yield strength of h-Gyroid and g-Gyroid implant structures comply with the strength requirements of the jawbone. Additionally, the Gyroid structure implant features multiple interconnected pores, which provide sufficient conditions for the inward growth of cells and tissues (41). The MIT values obtained in this study (approximately 19–23 N/cm) are consistent with previous reports for porous titanium implants in rabbit models. Notably, while the g-Gyroid implants exhibited slightly higher torque values, the absence of statistical difference implies that the gradient porosity structure does not negatively affect bone-implant interlock. This is likely due to the smooth surface transition and maintained outer stiffness of the g-Gyroid design, which ensures adequate friction at the cortical bone interface.

The interconnected pores in porous implants enhance material exchange with the external environment and facilitate the ingress of residual substances such as air and liquids, thereby compromising osteogenic efficacy at the implant center (42). Consequently, prior to animal experiments, we filled the pores of porous implants with Poloxamer 407 hydrogel. The Poloxam hydrogel implant served as a temporary scaffold after each Gyroid structure implantation, gradually degrading post-implantation. Histological staining revealed extensive bone and fibrous tissue infiltration within both h-Gyroid and g-Gyroid porous scaffolds, with the hydrogel degradation rate not impeding the growth of neo-tissue within the scaffold. Additionally, the maximum torque during implantation met clinical application requirements without statistically significant differences.

Meanwhile, histological staining revealed that bone tissue grew exclusively from one side of the Gyroid structure implant. Moreover, the average BIC% of g-Gyroid implants at 4 weeks was significantly higher than that of h-Gyroid implants. This may be attributed to the gradient variation of the Gyroid structure, where the g-Gyroid structure exhibited stronger bone induction capability in the early stage (4 weeks) within the 60%–80% gradient range compared to the h-Gyroid structure. Additionally, based on the average values of BV/TV, trabecular number, thickness, and trabecular spacing, no significant statistical differences were observed between h-Gyroid and g-Gyroid implants at 8 weeks post-implantation. This indicates that the edges of g-Gyroid implants possess significant bone integration capability, which may be enhanced by SLA processing. In animal experiments, the implants were placed into the femoral bones of rabbits. The neck region of the implant directly contacts the bone defect area, while the apical portion is typically exposed in the medullary cavity, away from the bone defect. Statistical analysis revealed that the BIC values of the neck regions of both h-Gyroid and g-Gyroid implants were higher than those of the apical portions, with no overall statistical difference. This suggests that g-Gyroid structure implants exhibit superior bone guidance effects, enabling more bone tissue to be incorporated into the Gyroid structure even when the apical portion is located in the medullary cavity, away from the bone fracture site.

Furthermore, the osteogenic effect of the Gyroid structure may also be attributed to the curved surface of its pores, which facilitates early adhesion and proliferation of bone tissue (43). This is because the curved surface directly contacts adjacent bone defect areas, and the Gyroid structure pores are interconnected. Additionally, scaffolds with porosity exceeding 50% have been demonstrated to exhibit high osteoconductivity (44). The two Gyroid-structured implants we utilized both had porosities ranging from 60% to 80%, providing a bone bed that enables bone tissue to grow into the bone from all contact surfaces (45). The fully interconnected Gyroid structure offers a larger surface area for new bone formation. Based on these findings, g-Gyroid-structured implants play a critical role in regulating bone tissue growth into the Gyroid structure.

## 5 Conclusion

This study digitally designed a gradient porosity Gyroid-structured porous implant and fabricated it using selective laser melting (SLM) technology. The elastic modulus of this gradient porosity Gyroid-structured porous implant matches that of cortical bone and cancellous bone, significantly reducing the stress shielding effect. Meanwhile, its strength also meets the requirements of bone implants. Additionally, the porous implant with a gradient porosity Gyroid structure exhibits excellent osteogenic capacity. Furthermore, this study provides new insights into the design and application of porous implants.

## Data Availability

The original contributions presented in the study are included in the article/Supplementary Material, further inquiries can be directed to the corresponding author/s.
