# Supplementary material for: Mechanical and biological properties of 3D printed bionic porous Gyroid structure implant
Source: Front Dent Med. 2026 Jun 2;7:1806825. doi: 10.3389/fdmed.2026.1806825 (PMC13269322; doi:10.3389/fdmed.2026.1806825)
Supplement: Supplementary file 1 [file Table1.docx]

Supplementary Material

# Supplementary Tables

**Supplementary Table 1**. Elastic Modulus, yield strength, compressive strength, and compressive strain of h-Gyroid and g-Gyroid structures before and after SLA.

| Group | Elasticity modulus  (GPa) | Yield  trength  (MPa) | Compressive  strength  (MPa) | Compressive  strain  (%) |
| --- | --- | --- | --- | --- |
| h-Gyroid | 3.43±0.31 | 167.29±7.31 | 233.56±2.35 | 15.42±0.55 |
| h-Gyroid-SLA | 3.55±0.08 | 150.01±2.14 | 216.43±2.83 | 15.38±0.34 |
| g-Gyroid | 3.38±0.13 | 152.14±2.70 | 205.98±0.13 | 10.97±0.73 |
| g-Gyroid-SLA | 3.21±0.17 | 127.49±1.41 | 169.42±2.47 | 9.40±0.39 |

# Abbreviations

The following abbreviations are used in this manuscript.

| abbreviation | full name |
| --- | --- |
| BITC | Beijing Implant Training College |
| TPMS | triply periodic minimal surface |
| SLM | selective laser melting technology |
| SLA | Sand-blasted, Large grit, Acid-etched |
| TC4 | titanium alloy |
| TA1 | Pure titanium |
| STL | the standard triangular language |
| CLS | Concept Laser Slicer |
| E | elastic modulus |
| σs | offset yield strength |
| σbc | compressive strength |
| h-Gyroid | 70% homogeneous porosity porous implants |
| g-Gyroid | 60%-80% gradient porosity porous implants |
| BIC | The bone-implant contact |
| MIT | the maximum implant insertion torque |
| BV/TV | Bone volume fraction |
